# Supplementary material for: Identification of Freezing-Responsive microRNAs and Their Targets in Chinese Jujube by Small RNA and Degradome Sequencing
Source: Int J Mol Sci. 2026 Sep 4;27(17):7912. doi: 10.3390/ijms27177912 (PMC13566890; doi:10.3390/ijms27177912)
Supplement: Supplementary file 1 [file ijms-27-07912-s001.zip › Supplementary Material S2/novel_miR_206.pdf]

Provisional ID	:	novel_miR_206
Score total	:	900.9
Score for star read(s)	:	3.9
Score for read counts	:	894.4
Score for mfe	:	1
Score for randfold	:	1.6
Score for cons. seed	:	
Total read count	:	1766
Mature read count	:	1748
Loop read count	:	0
Star read count	:	18

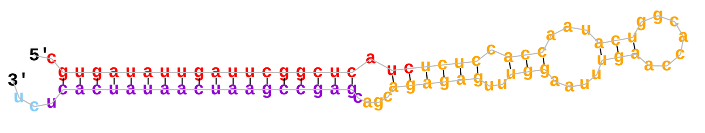

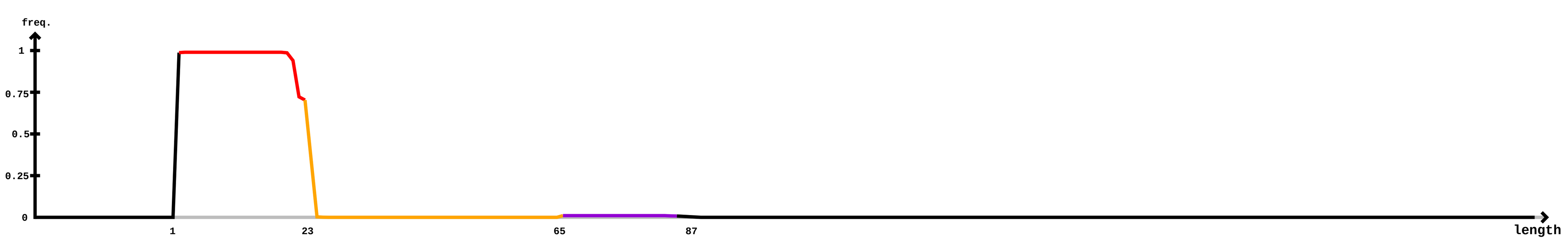

Mature Star

```
5'- cauucugcagaaguagacauggcgugauauugauucggcucaucucuccaccaauacuggcaccaaguuuaagguuugagagacgacgagccgaaucaauaucacucuuguaugcuucuucgcauauuaucaagguuuuuccuguuuuuugaguuccaauauuagccaagaagcaucucugcaggcaacucaugcauuaagcuacauagaagcuuguuuuggcucuccaucccccucuuguaaagucuuc  -3'  obs
    cauucugcagaaguagacauggcgugauauugauucggcucaucucuccaccaauacuggcaccaaguuuaagguuugagagacgacgagccgaaucaauaucacucuuguaugcuucuucgcauauuaucaagguuuuuccuguuuuuugaguuccaauauuagccaagaagcaucucugcaggcaacucaugcauuaagcuacauagaagcuuguuuuggcucuccaucccccucuuguaaagucuuc       exp
    ....(((((((.........((.((((((((((((((((((.((((((.(((...(((.......)))....)))..))))))....)))))))))))))))))).)).((((((......))))))......((.....))((((((((.(((..........)))))))))))..)))))))(((.....)))..((((((.......))))))....((((...((.........))...))))...   reads  mm  sample
    ......................cgugauauugauucggcu..........................................................................................................................................................................................................   1      0   LT-1
    ......................cgugauauugauucggcu..........................................................................................................................................................................................................   5      0   LT-3
    ......................cgugauauugauucggcuc.........................................................................................................................................................................................................   13     0   LT-3
    ......................cgugauauugauucggcuA.........................................................................................................................................................................................................   6      1   LCK-3
    ......................cgugauauugauucggcuc.........................................................................................................................................................................................................   7      0   LT-1
    ......................cgugauauugauucggcuc.........................................................................................................................................................................................................   21     0   LT-2
    ......................cgugauauugauucggcuc.........................................................................................................................................................................................................   33     0   LCK-3
    ......................cgugCuauugauucggcuc.........................................................................................................................................................................................................   1      1   LCK-3
    ......................cgugauauugauucggcuU.........................................................................................................................................................................................................   1      1   LT-3
    ......................cgugauCuugauucggcuca........................................................................................................................................................................................................   1      1   LCK-1
    ......................cgugauauugauucggcuca........................................................................................................................................................................................................   60     0   LCK-2
    ......................cgugauCuugauucggcuca........................................................................................................................................................................................................   1      1   LCK-2
    ......................cgugauauugauucgAcuca........................................................................................................................................................................................................   1      1   LCK-3
    ......................cgugauauugauucggcuca........................................................................................................................................................................................................   52     0   LT-3
    ......................cgugauCuugauucggcuca........................................................................................................................................................................................................   1      1   LT-3
    ......................cgugauauugauucggcuca........................................................................................................................................................................................................   3      0   JT-3
    ......................cgugauauugauucggcucU........................................................................................................................................................................................................   3      1   LT-2
    ......................cgugauauugauucggcuca........................................................................................................................................................................................................   23     0   LT-1
    ......................cgugauauugauucggcuca........................................................................................................................................................................................................   2      0   JCK-1
    ......................cgugauauugauucggcuca........................................................................................................................................................................................................   48     0   LCK-1
    ......................cgugauauugauucggcuca........................................................................................................................................................................................................   3      0   JCK-3
    ......................cAugauauugauucggcuca........................................................................................................................................................................................................   1      1   LT-2
    ......................cgugauauugauucggcuca........................................................................................................................................................................................................   1      0   JT-2
    ......................cgugauauugauucggcuca........................................................................................................................................................................................................   9      0   JCK-2
    ......................cgugauauugauucggcuca........................................................................................................................................................................................................   71     0   LT-2
    ......................cgugauauugauucggcuca........................................................................................................................................................................................................   103    0   LCK-3
    ......................cgugauauugauucggcucau.......................................................................................................................................................................................................   14     0   LCK-3
    ......................cgugauauugauucggcucaA.......................................................................................................................................................................................................   1      1   LT-3
    ......................cgugauauugauucggcucau.......................................................................................................................................................................................................   9      0   LT-2
    ......................cgugauauugauucggcucau.......................................................................................................................................................................................................   8      0   LT-1
    ......................cgugauCuugauucggcucau.......................................................................................................................................................................................................   1      1   LT-3
    ......................cgugauauugauucggcucau.......................................................................................................................................................................................................   1      0   LT-3
    ......................cgugauauugauucggcucauU......................................................................................................................................................................................................   5      1   LT-3
```

```
                      Mature                                                              Star
cauucugcagaaguagacauggcgugauauugauucggcucaucucuccaccaauacuggcaccaaguuuaagguuugagagacgacgagccgaaucaauaucacucuuguaugcuucuucgcauauuaucaagguuuuuccuguuuuuugaguuccaauauuagccaagaagcaucucugcaggcaacucaugcauuaagcuacauagaagcuuguuuuggcucuccaucccccucuuguaaagucuuc
......................cgugauauugauucggcucauc..............................................................................................................................................................................    237   0   LT-1
......................cgugauauugauucggcucauU..............................................................................................................................................................................    9     1   LCK-3
......................cgugauauugauucggcucauc..............................................................................................................................................................................    248   0   LT-3
......................cgugauauugauucggcucauc..............................................................................................................................................................................    31    0   LCK-1
......................cgugauauugCuucggcucauc..............................................................................................................................................................................    1     1   JCK-1
......................cgugauauugauucggcucauc..............................................................................................................................................................................    31    0   LCK-2
......................cgugauauugauucggcucauU..............................................................................................................................................................................    3     1   LT-1
......................Agugauauugauucggcucauc..............................................................................................................................................................................    1     1   LT-1
......................cgugauauugauucggcucauc..............................................................................................................................................................................    137   0   LCK-3
......................cgugauauugauucggcucCuc..............................................................................................................................................................................    1     1   LT-2
......................cgugauauugCuucggcucauc..............................................................................................................................................................................    1     1   LT-1
......................cguAauauugauucggcucauc..............................................................................................................................................................................    1     1   JCK-3
......................cgugCuauugauucggcucauc..............................................................................................................................................................................    1     1   LT-3
......................cgugauauugCuucggcucauc..............................................................................................................................................................................    1     1   JCK-2
......................cNugauauugauucggcucauc..............................................................................................................................................................................    1     1   JCK-1
......................cgugauauugauucggcucauc..............................................................................................................................................................................    122   0   LT-2
......................cgugauauugauucggcucauc..............................................................................................................................................................................    93    0   JCK-3
......................cgugauauugauucggcucauc..............................................................................................................................................................................    51    0   JCK-1
......................cgugauauugauucggcucauU..............................................................................................................................................................................    13    1   LT-2
......................cgGgauauugauucggcucauc..............................................................................................................................................................................    1     1   LT-2
......................cguUauauugauucggcucauc..............................................................................................................................................................................    1     1   LT-2
......................cgugauauugauucggcucauU..............................................................................................................................................................................    1     1   JCK-1
......................cgugauauugauucggcucauc..............................................................................................................................................................................    99    0   JCK-2
......................cgugCuauugauucggcucauc..............................................................................................................................................................................    1     1   LCK-3
......................cgugauauugauucggcucauU..............................................................................................................................................................................    1     1   JCK-2
......................cgugauauugauucggcucauU..............................................................................................................................................................................    5     1   JCK-3
......................cgugauauugauucggcCcauc..............................................................................................................................................................................    1     1   LT-1
......................cgugCuauugauucggcucauc..............................................................................................................................................................................    2     1   JCK-2
......................cgugauauugauucggcucaucu.............................................................................................................................................................................    30    0   LT-1
......................cgugauauugauucggcucaucA.............................................................................................................................................................................    1     1   LCK-3
......................cgugauauugauucggcucaucA.............................................................................................................................................................................    3     1   LT-3
......................cgugauauugauucggcucaucu.............................................................................................................................................................................    4     0   JCK-3
......................cgugauauugauucggcucaucu.............................................................................................................................................................................    3     0   JCK-2
......................cgugauauugauucggcucaucu.............................................................................................................................................................................    15    0   LCK-3
......................cgugauauugauucggcucaucA.............................................................................................................................................................................    3     1   LT-2
......................cgugauauugauucggcucauUu.............................................................................................................................................................................    6     1   LT-2
......................cgugauauugauucggcucaucu.............................................................................................................................................................................    1     0   JCK-1
......................cgugauauugauucggcucauUu.............................................................................................................................................................................    2     1   LT-1
......................cgugauauugauucggcucaucu.............................................................................................................................................................................    17    0   LT-2
......................cgugauauugauucggcucauAu.............................................................................................................................................................................    1     1   LT-2
......................cAugauauugauucggcucaucu.............................................................................................................................................................................    1     1   LT-2
......................cgugauauugauucggcucauUu.............................................................................................................................................................................    9     1   LCK-3
......................cgugauauugCuucggcucaucu.............................................................................................................................................................................    1     1   LT-1
......................cgugauauugauucggcucaucA.............................................................................................................................................................................    2     1   LT-1
......................cgugauauugauucggcucaucu.............................................................................................................................................................................    34    0   LT-3
......................cgugauauugauucggcucauUu.............................................................................................................................................................................    1     1   LT-3
......................cgugauauugauucggcucaucuU............................................................................................................................................................................    2     1   LT-3
......................cgugauauugauucggcucaucuU............................................................................................................................................................................    1     1   JCK-2
......................cgugauauugauucggcucaucuU............................................................................................................................................................................    1     1   LT-2
......................cgugauauugauucggcucaucuUu...........................................................................................................................................................................    1     1   LCK-3
.......................gugauauugauucggcuca................................................................................................................................................................................    1     0   LT-2
.......................gugauauugauucggcucauc..............................................................................................................................................................................    1     0   LT-1
.......................gugauauugauucggcucauc..............................................................................................................................................................................    2     0   LT-3
......................................................................................cgagccgaaucaauauca..................................................................................................................................................    1     0   LT-2
......................................................................................cgagccgaaucaauaucU..................................................................................................................................................    2     1   LCK-3
......................................................................................cgagccgaaucaauaucac.................................................................................................................................................    1     0   LT-1
......................................................................................cgagccgaaucaauaucacu................................................................................................................................................    5     0   LT-2
......................................................................................cgagccgaaucaauaucacu................................................................................................................................................    6     0   LT-1
......................................................................................cgagccgaaucaauaucacu................................................................................................................................................    2     0   LT-3
......................................................................................cgagccgaaucaauaucacuU...............................................................................................................................................    1     1   LT-1
```
